# Mitochondria–plasma membrane interactions and communication

**DOI:** 10.1016/j.jbc.2021.101164

**Published:** 2021-09-03

**Authors:** Pavel Montes de Oca Balderas

**Affiliations:** 1 Unidad de Neurobiología Dinámica, Department of Neurochemistry, Instituto Nacional de Neurología y Neurocirugía, Mexico City, Mexico; 2 Lab. BL-305, Instituto de Fisiología Celular, Department of Cognitive Neuroscience, Universidad Nacional Autónoma de México, Mexico City, Mexico

**Keywords:** plasma membrane, mitochondria, organelle, caveolae, mitochondria metabolism, metabolism, cell metabolism, evolution, organelle interactions, tunneling nanotubes, Cav-3, caveolin-3, CNS, central nervous system, Cx, connexins, EC, extracellular, ER, endoplasmic reticulum, ET, electron tomography, GJs, gap junctions, I/R, ischemia/reperfusion, iCa^2+^, intracellular Ca^2+^, IMM, inner mitochondrial membrane, IP, ischemia preconditioning, IS, immunological synapse, MAC, mitochondria-associated adherens complex, mCa^2+^, mitochondrial Ca^2+^, OMM, outer mitochondria membrane, PM, plasma membrane, SLP-2, somatostatin-like protein 2, TEM, transmission electron microscopy, TNTs, tunneling nanotubes

## Abstract

Mitochondria are known as the powerhouses of eukaryotic cells; however, they perform many other functions besides oxidative phosphorylation, including Ca^2+^ homeostasis, lipid metabolism, antiviral response, and apoptosis. Although other hypotheses exist, mitochondria are generally thought as descendants of an α-proteobacteria that adapted to the intracellular environment within an Asgard archaebacteria, which have been studied for decades as an organelle subdued by the eukaryotic cell. Nevertheless, several early electron microscopy observations hinted that some mitochondria establish specific interactions with certain plasma membrane (PM) domains in mammalian cells. Furthermore, recent findings have documented the direct physical and functional interaction of mitochondria and the PM, the organization of distinct complexes, and their communication through vesicular means. In yeast, some molecular players mediating this interaction have been elucidated, but only a few works have studied this interaction in mammalian cells. In addition, mitochondria can be translocated among cells through tunneling nanotubes or by other mechanisms, and free, intact, functional mitochondria have been reported in the blood plasma. Together, these findings challenge the conception of mitochondria as organelles subdued by the eukaryotic cell. This review discusses the evidence of the mitochondria interaction with the PM that has been long disregarded despite its importance in cell function, pathogenesis, and evolution. It also proposes a scheme of mitochondria–PM interactions with the intent to promote research and knowledge of this emerging pathway that promises to shift the current paradigms of cell biology.

Mitochondria have long been known as the energy-producing organelle of eukaryotic cells, through **oxidative phosphorylation** reactions that lead to ATP production (please find in Fig. 1 the general organization of mitochondria and in Table 1 a brief description of terms in the bold font used in this review). However, today it is well known that these organelles are also involved in other critical activities coupled to the dynamic intracellular conditions. Mitochondria are thought to have evolved from an α-proteobacteria that a long time ago adapted to intracellular living within an Asgard archaebacteria, the precursor of eukaryotic cells (1, 2). The origin of this association is not known, but it is proposed that it could have started as a symbiotic, predatory, or another kind of relationship (3). Be it as it was, what is clear today is that such association allowed the diversification of the primordial eukaryotic cell, which led to the appearance of multicellular organisms. There are several ideas regarding how the association between cell host and mitochondria evolved into such complexity and integration and how it has carved cell evolution to reach the actual complexity and diversity of eukaryotic cells (2, 3). This is intriguing mainly considering the diversity of host–mitochondria integration throughout the phylogenetic tree (4).

**Figure 1:**
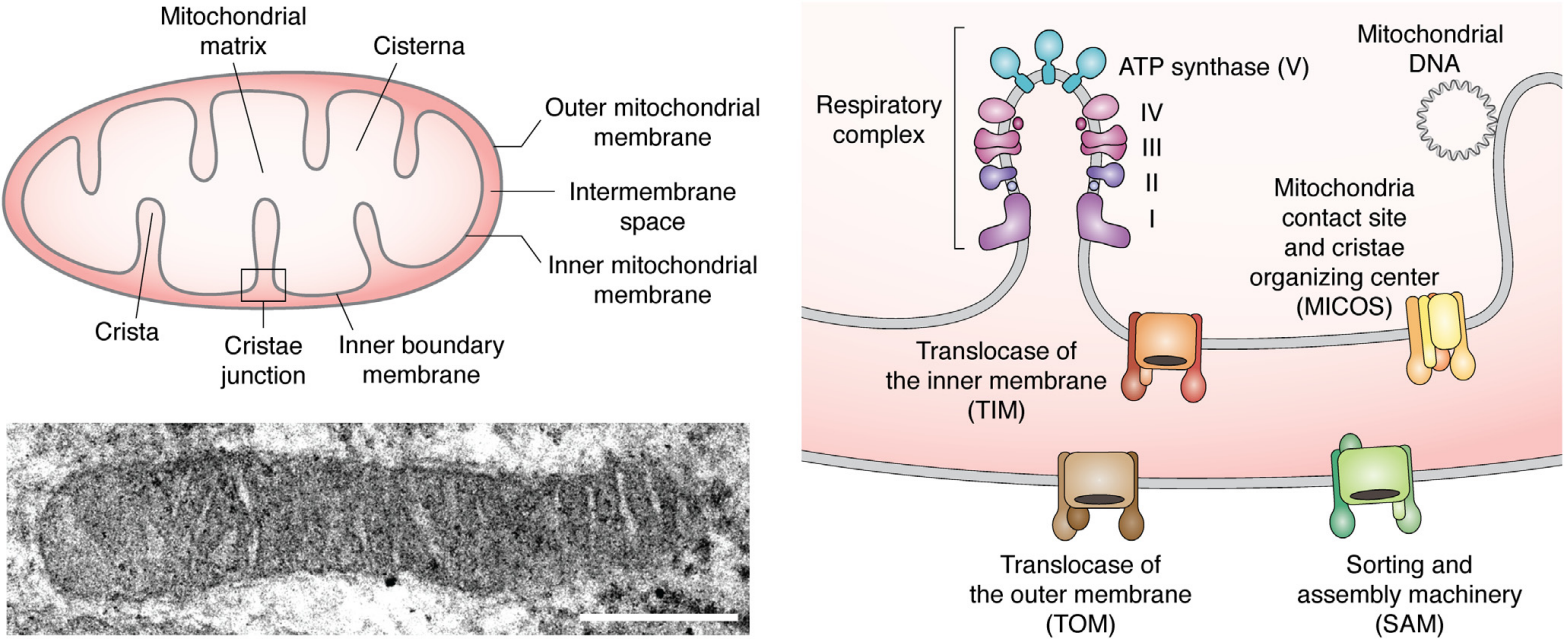
**General organization of mitochondria.** The mitochondrion is a cellular organelle characterized by a double membrane that performs oxidative phosphorylation, that results in the synthesis of ATP. In the *upper panel*, mitochondrial main structures and compartments are shown. These include the mitochondria matrix, surrounded by the inner mitochondria membrane (IMM), which in turn is surrounded by the outer mitochondria membrane (OMM). The space between these two membranes is the intermembrane space. The IMM forms a series of folds that constitute the cristae, which in turn establish the crista junctions (*black square*), narrow, tubular openings that attach the cristae to the inner boundary membrane (IBM). These folds also form the cisternae, flattened, IMM-bounded compartments. In the *lower panel*, a TEM micrograph of a mitochondrion from a cultured rat astrocyte is shown, in which these structures and compartments are observable (scale bar = 250 nm). In the *right panel*, a close-up and some important molecular players are depicted. These are the ATP synthase (the respiratory complex V) and the respiratory complexes I-IV located at or near the more inner folding of the IMM. The mitochondrial DNA (mtDNA) is also associated with the IMM. At the cristae junction (*black square*), several molecular complexes are located that mediate the transport of molecules to mitochondria. In the OMM, the translocase of the outer membrane (TOM) and the sorting and assembly machinery (SAM) are located, whereas in the IMM, the translocase of the inner membrane (TIM) and the mitochondria contact site and crista organizing center (MICOS) are located. TEM, transmission electron microscopy.

**Table 1:** Glossary of some cell biology terms in bold font used in this text

**Axo-dendrite:** contact between the axon of a neuron and a dendrite.
**Caveolae:** invaginations of the PM that bud off internally, thought to form from lipid rafts.
**Connexin:** protein subunit of gap junctions.
**Desmosome:** cell–cell junction usually formed between epithelial cells with dense plaques of protein into which intermediate filaments of the neighboring cells insert.
**Dynein:** ATP-dependent motor protein that moves cargo along microtubules.
**Gap junction:** cell–cell junction with a channel pore that connects neighboring cells cytoplasm. It allows the movement of molecules <5 kDa.
**Hemidesmosome:** cell junction that anchors an epithelial cell to the basal lamina.
**Innexons:** transmembrane protein that forms gap junctions in invertebrates.
**Oxidative phosphorylation:** a process in which ATP synthesis is achieved by electron transfer through the electron transport chain to molecular oxygen, involving the generation of a H^+^ gradient that is used by the ATP synthase.
**Presynaptic bouton:** specialized compartment of the axon at the synapse that contains neurotransmitter vesicles.
**Puncta adherens:** protein complex that occurs at cell–cell junction in synapses.
**Subplasmalemmal:** refers to the molecules, events, or organelles that occur or are located beneath the PM.
**Subsarcolemmal:** refers to the molecules, events, or organelles that occur or are located beneath the PM of muscle cells.

In the last decades, the knowledge about the integration of mitochondria into the eukaryotic cell has reached an unexpected level. The picture of the mitochondria as an organelle suspended in the cytoplasm with the role of producing ATP has been modified considerably. Today it is well known that mitochondria participate in different roles within cell physiology such as ion buffering, antiviral response, lipid metabolism, apoptosis, or cell division, among others. Moreover, it has been extensively demonstrated in the last 2 decades that mitochondria are capable of interacting directly with other intracellular organelles (5, 6, 7, 8). The most studied interaction of this kind is perhaps that of mitochondria with the endoplasmic reticulum (ER), interaction that is critical for Ca^2+^ homeostasis, and lipid metabolism among other functions (9). The integration of mitochondria to the cell is so critical and its role a vital factor that many pathologies are known to be associated with or have a causal relationship with mitochondria dysfunction.

This review is an attempt to bring into attention the interaction of mitochondria with the plasma membrane (PM), which has been only slightly investigated but could have tremendous implications for mitochondria and cell physiology. This interaction challenges the extended conception considering mitochondria as obligate, subdued organelles of the eukaryotic cell, subjugated to cell physiology and the intracellular environment with scarce or no interaction with the extracellular (EC) milieu. This conception is dominant because the cell host is thought of as an interphase that keeps mitochondria isolated and protected from the external milieu. However, this could be not true for all mitochondria. Since the middle of the 20th century, some reports have shown the interaction of mitochondria with certain PM domains of mammalian cells, mainly adhesion sites. The interaction of the PM with mitochondria has been already described at the molecular level in yeast, and it is involved in their segregation during cell division (10, 11). Interestingly, some mitochondria have a relationship with the EC milieu that allows them to sense and respond to EC events. In this regard, the **subplasmalemmal** mitochondria have been known for some decades (12). These mitochondria are known to have chemical interactions with the PM, mediated by solutes that diffuse through the cytoplasm that regulate mitochondria or PM functions. Nonetheless, there is also evidence indicating that the interaction of mitochondria with the PM may also involve other means, including physical ones, that allow their sensing of the EC milieu that drives their physiological adaptation. Furthermore, free, intact, functional mitochondria have been reported recently in human blood (13). Moreover, it has been shown and revised elsewhere that mitochondria can be translocated among cells by the so-called tunneling nanotubes (TNTs), suggesting PM–mitochondria communication (14, 15). These findings documenting mitochondria–PM interactions open a new horizon for the study of mitochondria in cells, tissues, and other contexts. Therefore, the findings accumulated in this decade make the review of this kind of communication pertinent, to set up a new framework in which these interactions are considered when mitochondria and the eukaryotic cell physiologies are investigated, to achieve a more integrative understanding of this organelle and the cell.

Here, the literature that has studied or suggested the interaction between mitochondria and the PM is reviewed, first briefly summarizing the main recent findings regarding the attachment of mitochondria to the PM in the context of yeast cell division. Then, the literature that supports or has studied the interaction between mitochondria and the PM in mammalian cells at the morphological and functional levels, which are poorly understood, is reviewed. Afterward, some aspects of TNT that mediate mitochondria transfer are briefly examined in the context of mitochondria–PM interactions because this topic has been extensively reviewed elsewhere. Finally, some insights are discussed, and conclusions are drawn.

## Mitochondria–PM interactions

The contacts between mitochondria and other intracellular organelles (*i.e.*, ER) were described more than 50 years ago, and their functional implications have been already described in some cells and tissues and are currently under intense investigation worldwide (16). Nevertheless, the relationship between mitochondria and the PM has been only scarcely investigated or dismissed. However, several observations suggesting a specific interaction between mitochondria and the PM have been made since almost 70 years ago by EM in epithelial and neuronal cells. In those images, mitochondria were located very close to the PM, and strikingly, some of these mitochondria appeared to contact intercellular adhesion sites on the PM with reciprocally located mitochondria in the neighboring cell. However, and although the interaction of mitochondria with the PM could have enormous implications for cell and mitochondria physiology, these interactions have not been deeply investigated. Only in the last decade, the attachment of mitochondria to the PM that occurs during cell budding in yeast has been studied at the molecular level, and to my knowledge, only a few reports have demonstrated its functional induction by stressors in mammalian cells, but no molecular players mediating this interaction have been identified (10, 17, 18). In this section, the literature that has studied or suggested the interaction between mitochondria and the PM is reviewed.

### Mitochondria–PM interaction in yeast

The interaction between the PM and mitochondria in yeast was described a few years ago. Here, we will only summarize the main findings of this interaction; for further details, please refer to published reviews and references therein (10, 11).

**Figure 2:**
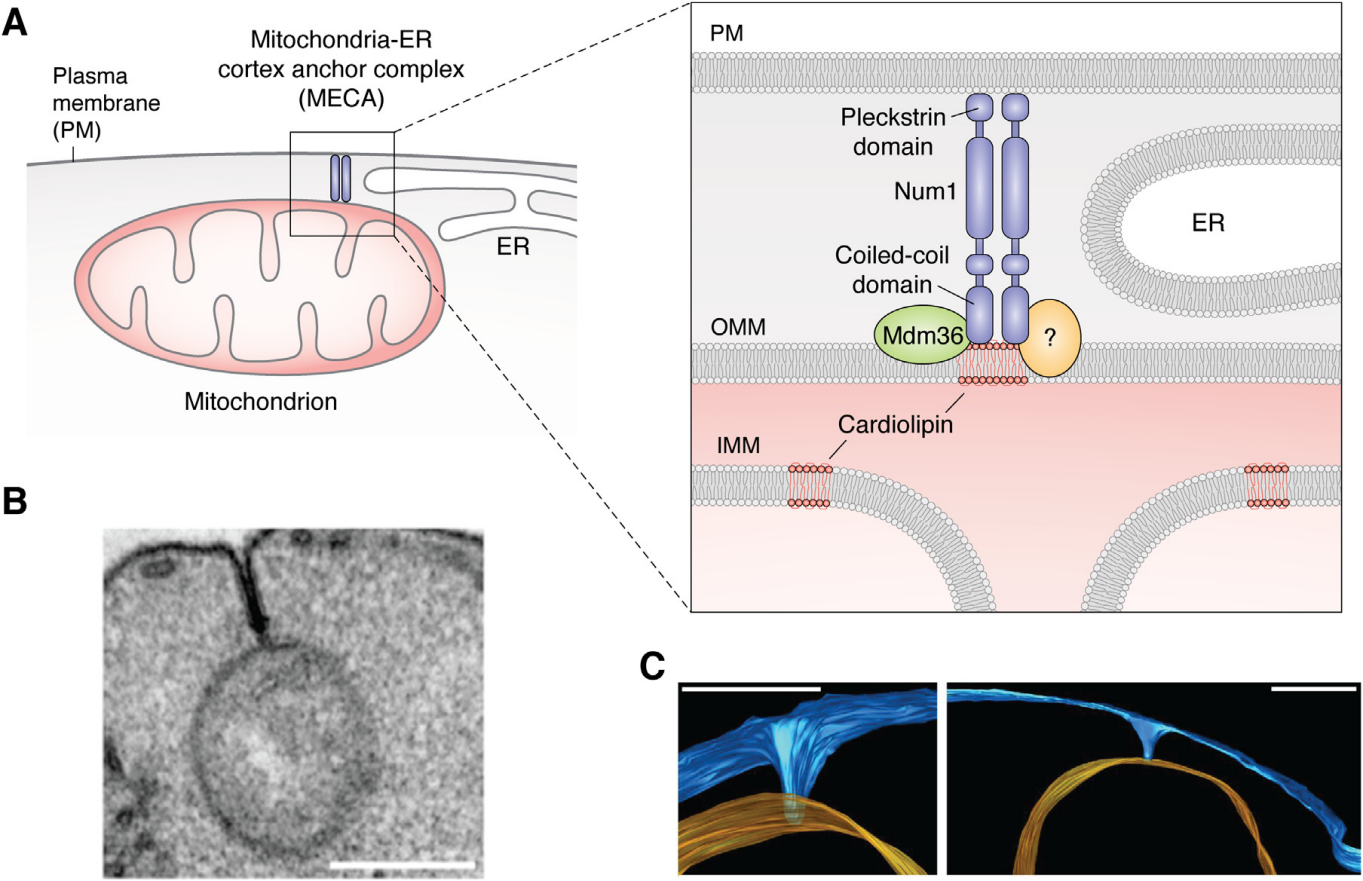
**PM–mitochondria interactions in yeast.***A*, the mitochondria–ER cortex anchor (MECA) complex tethers mitochondria, ER, and PM in yeast. In the *red square*, a close-up is shown. In this complex, Num1 (*purple*) binds to PM through its pleckstrin domain and to the OMM through its coiled-coil domain that interacts with cardiolipin (in *red*) or other negatively charged, cone-shaped phospholipids enriched at the cristae junction. The oligomerization of Num1 increases its avidity. Mdm36 in the OMM and most probably other unknown molecules (*yellow, question mark*) bind to Num1. The MECA also involves dynein that mediates PM anchoring (not shown) (19). *B*, TEM micrograph of the invaginations observed by Klecker *et al*. (21) that allow PM–mitochondria contact. The scale bar represents 250 nm. *C*, electron tomography reconstruction of these invaginations. In *blue* is the PM, in *orange* the OMM. The scale bar represents 250 nm (21). All figures are used with permission. ER, endoplasmic reticulum; OMM, outer mitochondria membrane; PM, plasma membrane; TEM, transmission electron microscopy.

During yeast cell division, intracellular organelles must be shared by the mother and daughter cells. Defects in this partition of organelles may generate defective cells, for instance, without mitochondria that eventually do not survive. It has been found in daughter cells that some mitochondria establish a tether with the PM during yeast cell division, mediated by the molecules Num1 and Mdm36 (Fig. 1) (19). This tether is normally associated with the ER, and therefore, these authors termed it the mitochondria–ER cortex anchor. The findings indicate that Num1 is attached to the PM through its pleckstrin homology domain, and to mitochondria phospholipids through its coiled-coil domain, mainly to cardiolipin and other negatively charged, cone-shaped phospholipids that may be enriched at crista junctions (Fig. 2*A*) (20). Num1 also attaches to Mdm36, which locates in the outer mitochondria membrane (OMM). It is proposed that other molecules beyond Num1/Mdm36 are involved in the PM interaction with mitochondria (19). In a different almost simultaneous study, Num1 was also identified as responsible for mitochondria retention in mother cells (21). Interestingly, in this work, the mitochondria–PM contacts were observed by transmission electron microscopy (TEM) and electron tomography (ET) (Fig. 2, *B* and *C*). It was found that PM invaginations spanning hundreds of nanometers contacted the OMM of mitochondria located 200 to 300 nm from the PM plane. Although these structures were also observed in Num1-deleted mutants, their interaction with mitochondria was not observed. These findings let the authors conclude that such structures are the Num1 mitochondria–cortex anchors, which integrate the mitochondrial dynamics into cellular architecture. More recently, it has been shown that for mitochondria–ER cortex anchor formation, Num1 assemblies into clusters that increase its avidity for partners (22). Some of these clusters also anchor **dynein** to the cell cortex; therefore, when Num1 function is altered, mitotic spindle orientation problems emerge. According to these authors, the fact that mitochondria are required for the assembly of Num1 anchoring clusters represents a mechanism that spatially and temporally controls tether function, a mechanism that could extend to other proteins that function to establish, maintain, and alter contacts between organelles.

Together, these findings demonstrate that mitochondria are capable of establishing functional interactions with the PM in yeast, and therefore that they may also occur in mammalian cells. In addition, the fact that PM invaginations mediate these tethers suggest a role of the cytoskeleton. Moreover, these findings further indicate that these interactions have dynamic and temporal features that are critical for their function.

### Mitochondria–PM interactions in mammalian cells: Morphologic evidence

#### Non–central nervous system cells

**Figure 3:**
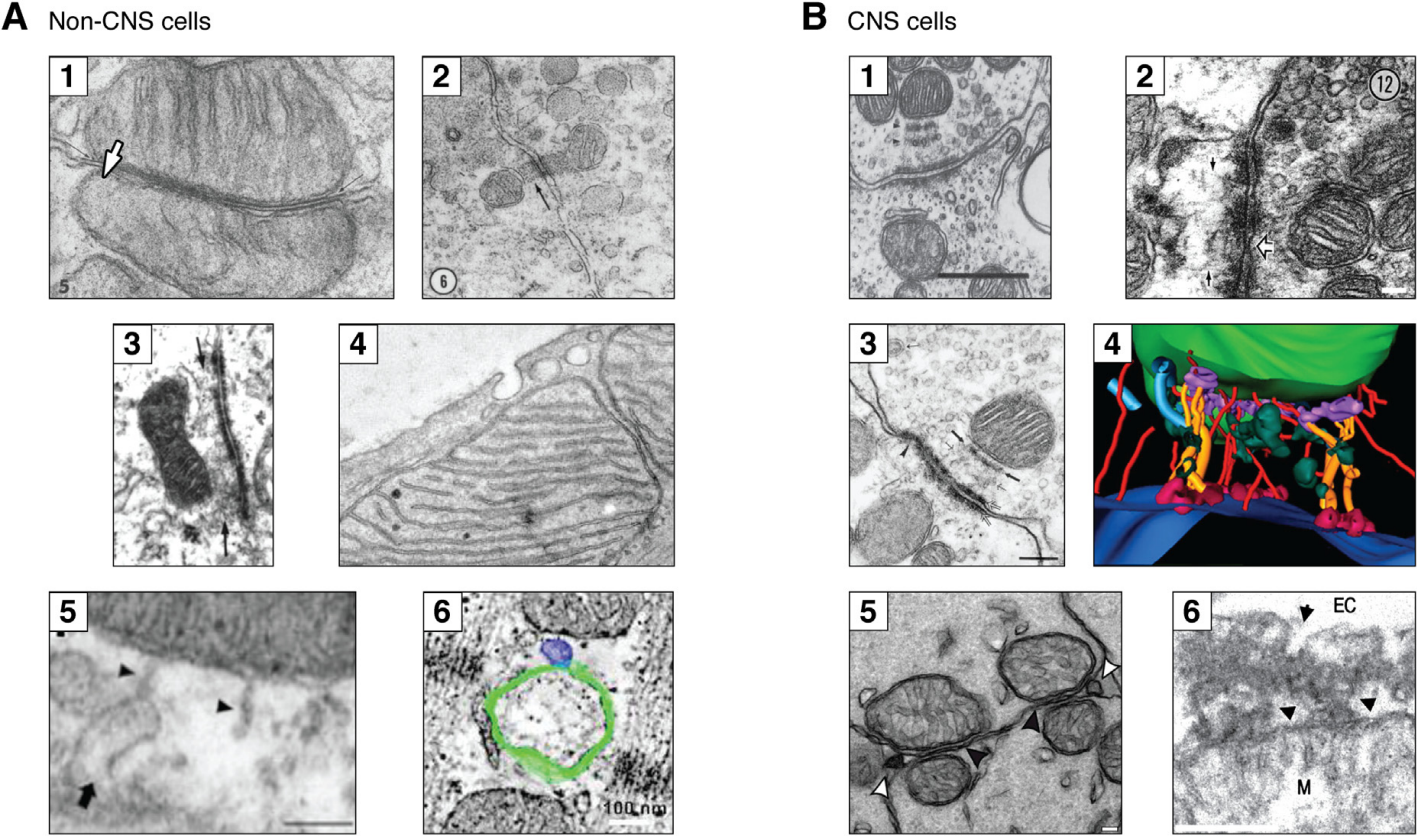
**PM–mitochondria interactions in non-CNS and CNS cells.***A1*, Fig. 5 from plate 4 in (28) showing the mitochondria-associated junction described by the author in follicular cells of the bat thyroid during late hibernation. Here, two mitochondria are shown from neighboring cells apposed to the PM with highly electrodense regions in between (the mitochondria-associated junction), with their cisterna oriented perpendicular to the PM plane. The *arrows* point to the close association between the OMM and the PM, X140,000. *A2*, Fig. 6 from (30) showing two mitochondria with fibers (*arrow*) on two sides of a desmosome in neighboring cells from the rat thyroid gland, X50,000. *A3*, Fig. 4 from (31) showing a mitochondrion of a human Sertoli cell adjacent to a spermatocyte. In this TEM micrograph, the filamentous material (*arrows*) of the desmosomes seem to contact mitochondria, X42800. *A4*, Fig. 1*B* from (18) showing cardiac myocyte mitochondria closely apposed to caveolae (scale not specified). *A5*, Fig. 1*F* from (18) showing evidence of increased association of caveola–mitochondria after ischemic preconditioning. Note the tubular extension that contact mitochondria (*arrowhead*), one of them apparently evaginated from the caveolae (*arrow*). The scale bar represents 50 nm. *A6*, segment of Fig. 4 in (35) showing an ET image of a T-tubule (*green*) membrane with an invaginating caveolae (*blue*), near a mitochondrion in rabbit ventricular tissue. The scale bar represents 100 nm. All figures were used with permission. *B*, PM–mitochondria interactions in CNS cells. *B1*, section of Fig. 9 in (27) showing the attachment plaque in the anteroventral cochlear nucleus of the cat. In the ending, a row of vesicles (*arrowheads*), overlying dense plaque, and filamentous material extending to mitochondria. The scale bar represents 500 nm. *B2*, section of Fig. 12 in (37) showing part of a symmetrical filamentous contact between an axon and a dendrite associated with mitochondria in the thalamic relay nuclei of rats. The *white arrow* shows a spot-like close membrane interaction suggested to be a GJ. The scale bar represents 100 nm. *B3*, Fig. 8*A* from (38) showing details of the mitochondria-associated adherens complex (MACs) in the lateral nucleus of the trapezoid body of the cat. This assembly is composed of the punctum adherens (*open arrows*); a mitochondrion often with its side facing the PM flattened and cristae oriented perpendicular to the PM plane; the mitochondria plaque (*small solid arrows*); the vesicular chain (*dotted arrows*) filamentous bands (f) form the puncta adherens to the mitochondrion; and often an associated mitochondrion in the postsynaptic cell. The scale bar represents 200 nm. *B4*, Fig. 5*D* from (40) showing an ET reconstruction close-up of the MAC in the Calyx of Held of the cat. In *dark blue*, is the presynaptic membrane; the mitochondria (*green*); microtubules (*blue*); microfilaments (*red* filaments); struts (*gold*); the mitochondrial plaque (*purple*); and punctum adherens (*light red* on the presynaptic membrane). *B5*, Fig. 1*E* from (43) showing the organization of mitochondria in photoreceptors of the mouse retina. Mitochondria are arranged in aligned (*black arrowheads*) doublets or triplets between neighboring inner segment regions of photoreceptors. *White arrowheads* point to membrane projections observed between the inner segments near mitochondria. The scale bar represents 100 nm. *B6*, Fig. 6*B* in (17) showing the PM–mitochondria bridges in cultured rat astrocytes. This structure is related to endocytic vesicles, most probably, caveolae for their size (*arrow*). It also comprises an electrodense area that seems to connect mitochondria with the PM that coincides with *dark spots* located within mitochondria. The perpendicular organization of mitochondria cisternae relative to the PM is also observed. The scale bar represents 250 nm. All figures are used with permission. EC, extracellular; ET, electron tomography; GJs, gap junctions; M, mitochondria; PM, plasma membrane; TEM, transmission electron microscopy.

The first observations that suggested an interaction of mitochondria with the PM were perhaps those made by Yamada and Palade by TEM (23, 24). The first work by Palade showed in the intestinal epithelium mitochondria located near the PM with **caveolae**-like vesicles in between. On the other hand, Yamada showed in mouse columnar epithelium of the gall bladder mitochondria that were located a few hundred nanometers (∼200–300) from the lateral wall, where electrodense structures spanning adjacent cells were observed, these were intercellular adhesion contacts. Later studies found further evidence of mitochondria–PM interaction associated with intercellular contacts in other cells and tissues including those of the central nervous system (CNS) (see below). Several groups observed mitochondria (sometimes with ER) located near intercellular contact sites (**desmosomes** or **puncta adherens**) in different cell types, mainly epithelial or neuronal cells from different tissues and species, but also in transformed cells, in which these interactions were frequent. Some authors reported mitochondria contacting multiple desmosomes, or mitochondria contacting intercellular adhesion sites (Fig. 3, *A1–A3*). Distances of mitochondria to the PM ranged from ∼300 nm up to ∼50 nm, and one study reported that half of the desmosomes have at least one associated mitochondria, although variation from cell to cell was observed (25, 26, 27, 28, 29, 30, 31, 32, 33). Interestingly, one report described in follicular cells of the bat thyroid under hibernation, large contact areas (as large as mitochondria itself) between mitochondria and the PM with a distance <10 nm, with striking specular mitochondria in the opposing cell (Fig. 3*A1*) (28). These structures were called organelle-associated intercellular junctions by the author because the ER could also be observed. Similar dispositions, with mitochondria on both sides of the intercellular adhesion site, although with larger gaps between mitochondria and the PM, were observed in rat thyroid cells (30) (Fig. 3*A2*), epithelial cells of the mouse seminal vesicle (25), human Sertoli cells (31) (Fig. 3*A3*), in rabbit eye epithelia (29), and hepatoma (26).

In cardiac myocytes of different species, one study found close apposition (∼10 nm) of mitochondria to PM **gap junctions** (GJs), thought previously to present a random distribution, with aligned opposing mitochondria from two different cells and supported the role of mitochondria for Ca^2+^ buffering (34). More recently, in cardiac myocytes, caveolae were observed near **subsarcolemmal** mitochondria and they were found critical for cellular health (see below) (Fig. 3*A4*) (18). Intriguingly, some caveola presented tubular extensions that contacted mitochondria (Fig. 3*A5*), resembling those invaginations occurring in yeast (Fig. 2*B*). Interestingly, in a recent article, Burton and cols. (2017) provided evidence obtained by TEM, scanning electron microscopy, and ET, indicating that caveolae are formed in cardiac myocytes T-tubules, near mitochondria located <100 nm from T-tubule membrane (Fig. 3*A6*) (35). Strikingly, these caveolae are abundant but decrease on time in culture, that according to the authors may have implications for work made with these cell models. They also reported caveolae that were located near mitochondria at the PM, consistently with earlier reports (18), and they also found a size increase of these subsarcolemmal mitochondria.

#### CNS cells

Since 1963, Gray observed in **presynaptic boutons** of the spinal cord mitochondria related to symmetric **axo-dendritic** attachment plaques (36). He also observed two striations and an electrodense area between the mitochondria and PM. Interestingly, in the images of this work, the cisternae of these mitochondria were oriented perpendicular to the plane of the PM, a common phenotype observed in other cells. The year after, similar structures were found in the lateral geniculate nucleus of the monkey (32). Two decades later, EM studies also observed mitochondria–intercellular adhesion site co-occurrence with filamentous elements at the interphase between, that were oriented perpendicular to the PM plane and other details. One report described a chain of vesicles and a dense plaque, both parallel to the PM in axo-dendrite synapses of the anteroventral cochlear nucleus of the cat (Fig. 3*B1*) (27). Similar observations were made by Spacek (1985), showing puncta adherentia and associated mitochondria in the mouse visual cortex (33). Later, mitochondria were also located by serial TEM near filamentous intercellular adhesion sites in the presynaptic bouton of thalamic relay nuclei of rats (37). In this work, the ER was identified as the chained vesicles observed at the interphase, and importantly, putative GJs were observed in the vicinity (Fig. 3*B2*). The presence of the ER led the authors to speculate about mitochondria function on synaptic plasticity and Ca^2+^ metabolism. The following year, one group studied mitochondria located near intercellular adhesion sites in the lateral nucleus of the trapezoid body of the cat (Fig. 3*B3*) (38). These authors observed a very similar organization to that reported earlier (27, 36), but in addition, they described the flattening of the mitochondrial surface oriented to the PM and the orientation of mitochondrial cisternae perpendicular to the PM, also captured in earlier works. With these elements, they named this interphase the mitochondria-associated adherens complex (MAC), to which mitochondria was tethered as suggested by the mitochondria membrane flattening. The MAC was preferentially located adjacent to areas of enlarged interstitial space and sites of presumed endocytosis. These observations let the authors hypothesize that the MAC could be involved in maintaining intercellular contacts at sites with extensive PM recycling, presumably because of the high rates of synaptic activity. In a different work, the same group further described the context of MAC at the presynapse through serial TEM (39). They found that MAC and some synapses form clusters, with MACs in the core and synapses surrounding them, with an average distance of 200 nm from MAC to the synapse, and an average distance of 1000 nm from synapses to MACs, average enlarged by synapses that do not have associated MACs. These structures were located near enhanced EC spaces, and sites of endocytosis with coated vesicles near to or fused to vesicle chains. Notably, in contrast to previous work (37), these vesicle chains were not identified as ER. It was also found that MAC mitochondria tend to be more complex, and its distance from the PM (200 nm) was a strong predictor of MAC formation. These authors hypothesized that MAC could be involved in the fast formation of vesicles, Ca^2+^ regulation facilitated by cristae orientation, glutamate synthesis for vesicle refilling, synapse stabilization, and formation. In a more recent article, the same group performed ET to study the MAC (Fig. 3*B4*) (40). They observed a higher density of cristae membrane, that according to the authors implicates higher metabolic activity and mitochondria polarization with a higher density of crista junctions located near the mitochondria plaque. They confirmed that the filamentous structures are cytoskeleton elements, mainly microtubules, that anchor the mitochondria to intercellular adhesion sites and observed that synaptic exocytic vesicles are excluded from these attachment sites. More recently, a different group found that mitochondria, at the same synapse studied by Spirou and Perkins (the Calyx of Held), have increased density and larger volumes, relative to terminal volume, after synapse maturation (p21) in comparison with earlier stages (p7) before hearing onset (p12) (41). Indeed, it has been shown that mitochondrial volumes are larger at high-activity synapses (42). These observations suggested a link between mitochondria localization and its function with synapse maturation when a high-fidelity synaptic transmission is established, accompanied by a reduction of neurotransmitter release probability. Unfortunately, these authors did not observe MACs, according to the authors, probably due to the protocol of EM used; nonetheless, MACs were proposed to mediate the increase of volume observed.

More recently, mitochondria were also found apposed (∼10 nm) to the PM in mouse photoreceptors, with mirroring mitochondria in the adjoining cell (Fig. 3*B5*), and some functional features were described (see below) (43). In astrocytes, mitochondria were also observed close (∼150 nm) to **hemidesmosome** contacts in perivascular processes, whereas mitochondria of the perisynaptic astrocyte projection were located even closer (∼50 nm) to the PM that contacts dendrites (44), similar to cultured astrocytes (17). In these same cells, a different group observed vesicles that could be caveolae between mitochondria and the PM. In addition, end-feet mitochondria were located near the PM facing the basal lamina, with an electrodense region between the PM and mitochondria, with a slight flattening of mitochondria membrane (45). More recently, in cultured astrocytes, we described the PM–mitochondria bridges as electrodense regions induced by the acidic N-methyl D-aspartic acid receptor (NMDAR) agonist related with small vesicles, most probably caveolae (see below) (Fig. 3*B6*) (17).

Taken together, these observations in non-CNS and CNS cells substantiate that mitochondria are capable to establish specific interactions with the PM, particularly at intercellular adhesion sites, sometimes mediated by the cytoskeleton. In line with these findings, MAC mitochondria were hypothesized to play specific roles, some of them already confirmed, as Ca^2+^ buffering (12). These observations lead to the idea that mitochondria might influence the formation or multiplication of intercellular contact sites or conform domains of intensive intercellular exchange (31). However, the existence of intercellular bridges that occur in the same cells as desmosomes cast doubts for the last assumption. Moreover, some authors considered that this association was probably without functional significance (26).

### Mitochondria–PM interactions in mammalian cells: Functional evidence

**Figure 4:**
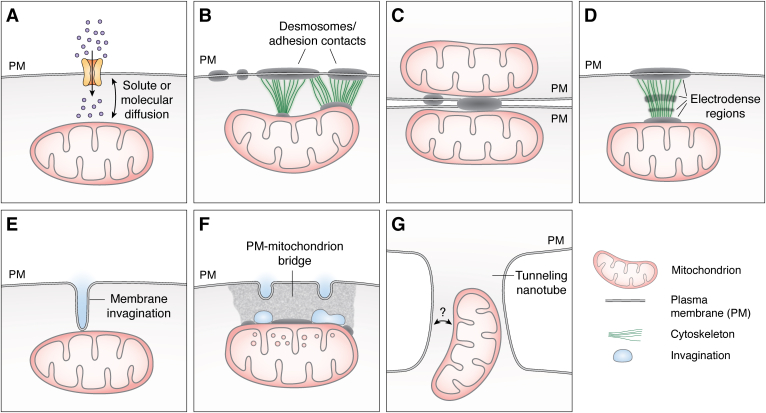
**Complexity levels of plasma membrane–mitochondria interactions.***A*, the most fundamental communication between the PM and mitochondria is mediated by the diffusion of solutes including ions (*i.e.*, Ca^2+^ or Na^+^), second messengers (*i.e.*, InsP3), or proteins (*i.e.*, signal transducers). This communication is bidirectional, indicated by the *double-headed arrow*. In *orange*, an ion-permeable channel. *B*, early TEM observations mostly in epithelial cells showed the interaction of mitochondria with PM domains such as desmosomes or other adhesion contacts as GJ or puncta adherens. These interactions presented filamentous arrangements (*green*) between desmosomes and mitochondria, highly electrodense regions at the PM, which may include the PM of the neighboring cell, and cisternae perpendicular to the PM plane. These interactions may comprise other mitochondria in the adjoining cell and different distances to the PM (Fig. 3, *A2* and *A3*) [drawing based on (31)]. *C*, mitochondria tethered to the PM in two neighboring cells with highly electrodense regions in between, with their cisterna oriented perpendicular to the PM plane have been reported. This disposition of mitochondria seems to be related to cellular synchronization (Fig. 3, *A1* and *B5*) [drawing based on (28)]. *D*, more elaborated structures have been observed in presynaptic neurons in several CNS regions (Fig. 3, *B1–B4*), with several bands between the PM and mitochondria, filamentous structures (*green*), highly electrodense regions at the PM of presynaptic and postsynaptic neurons, flattening of the mitochondria membrane that faces the PM, and perpendicular cisternae to the PM. Occasionally, mitochondria in the postsynaptic cell can be observed close and in front of these structures (Fig. 3, *B1–B3*) [drawing based on (36)]. These structures were observed later by different groups and were named mitochondria adherens complex (MAC) in (38), the group that also did ET of these structures (40) (Fig. 3, *B3* and *B4*). *E*, membrane invaginations (*blue*) extending into the cytoplasm that contact mitochondria have been described in yeast PM (21) and caveolae from myocytes (18). In the case of yeast, some molecular players of the MECA complex have been elucidated (19) [drawing based on (21)]. *F*, caveolae have been implicated in PM–mitochondria interactions (Fig. 3*A4*, *A5*, and *B6*). In cultured astrocytes, we described the PM–mitochondria bridges, consisting of a highly electrodense region between the PM and mitochondria, which is associated with invaginated vesicles (*blue*) with the size of caveolae, flattening of the mitochondria membrane facing the PM, and *dots* within mitochondria that also present cisternae perpendicular to the PM. These structures mediated the mass transfer from the PM to mitochondria in minutes [drawing based on (17)]. *G*, tunneling nanotubes (TNTs) are cellular structures that have been shown to mediate cargo transfer between cells, including mitochondria. The interaction between the PM and mitochondria has not been demonstrated, but it probably occurs (*question mark*) as mitochondria are located at the TNT entry and trespass the PM plane. ET, electron tomography; PM, plasma membrane; TEM, transmission electron microscopy.

For decades, several groups have demonstrated or inferred a functional interaction between the mitochondria and PM, but only a few have demonstrated that these interactions can be physical and instrumental. It has been demonstrated that subplasmalemmal mitochondria are regulated by PM molecules and events and that PM molecules are regulated by mitochondria, implicating crosstalk between these organelles (Fig. 4*A*). The control of intracellular Ca^2+^ (iCa^2+^) levels by mitochondria is perhaps one of the most studied mechanisms, and it is known since the 60s (12). Here, we briefly review some of these events that enrich the matter of our subject, and then those that have revealed functional–physical interactions.

#### Nonphysical interactions

In rat-cultured striatal neurons, it was found that iCa^2+^ and mitochondrial Ca^2+^ (mCa^2+^) rise were coordinated in response to NMDAR activation (46). In contrast, other stimuli showed a delayed rise of mCa^2+^ relative to iCa^2+^. These authors proposed that Ca^2+^ flux through the NMDAR, a receptor that is critical for synaptic communication and neuronal health, has privileged access to mitochondria.

In T cells, it was found that their activation and the establishment of the immunological synapse (IS), a special intercellular contact site of immune cells, generated the approximation of mitochondria to the IS (<200 nm), dependent upon the microtubule and actin cytoskeleton (47). At the IS, mitochondria buffer Ca^2+^ entry from ORAI1 channels, maintaining Ca^2+^ levels low, which in turn regulates ORAI1 and PM-Ca^2+^ ATPase activities, mechanisms critical for proper T cell activation. Although Ca^2+^ uptake by mitochondria at ORAI channel domains had been observed previously by different groups, the dynamic nature of mitochondria in this mechanism was evidenced for the first time. Moreover, these authors confirmed that this Ca^2+^ buffer function of these mitochondria with ORAI1 channels is cell dependent because it was not observed in HeLa cells. In this regard, it is relevant to recall the so-called mitochondria firewall that protects secretory cells from an excess entry of Ca^2+^ at the apical membrane (the secretory side), and its diffusion to the basolateral membrane (48). This is a clear example of the mitochondria–PM interaction relevance, a function that is very important in different cells and tissues (12).

In cultured astrocytes, it was observed by total internal reflection fluorescence microscopy that glutamate or ATP induces the approximation and trapping of mitochondria near the basal PM (49). According to these authors, mitochondria trapping depended upon iCa^2+^ rise, which could be from intracellular or EC sources and involved microtubule activity. These authors suggested that this mechanism could be relevant for Ca^2+^ uptake by mitochondria, in agreement with previous findings (46), or for the regulation of transmitter release.

#### Physical interactions

Regarding the functional and physical interactions, in cardiac myocytes, a subset of PM caveolae localize near subsarcolemmal mitochondria. Interestingly, the number of mitochondria-associated caveolae increased after ischemia/reperfusion (I/R) or increased transiently after oxygen/glucose deprivation, suggesting the dynamic communication between mitochondria and the PM (Fig. 3*A4*) (18). Consistently with this idea, the authors found the transfer of caveolin-3 (Cav-3) from the PM to the inner mitochondrial membrane (IMM) of these mitochondria, a small scaffolding protein fundamental for the formation of caveolae (50). These authors further confirmed that Cav-3 overexpression protects myocytes from I/R and found mitochondria with higher respiratory rates that tolerated higher Ca^2+^ concentrations and generated less reactive oxygen species, while loss of Cav-3 promoted mitochondria dysfunction. These observations implicated a role of Cav-3 in cell adaptation to stress, through maintenance of mitochondrial function. Interestingly, although not described by the authors, the cisternae from these mitochondria tend to be oriented perpendicular to the PM, as described in presynaptic mitochondria (see above) and observed in earlier reports. However, the authors did not find a structural arrangement such as MACs at the interphase between the PM and mitochondria. Nevertheless, these authors identified tubular invaginations from caveolae to mitochondria after I/R (Fig. 3*A5*).

Interestingly and in addition to Cav-3, different groups have reported other PM molecules located at the mitochondria, or mitochondria molecules located at the PM, suggesting their interaction and bidirectional mass exchange. Functional NMDARs have been described at the IMM of mitochondria isolated from the cerebral cortex of rats. These receptors mediated the rise of mCa^2+^, promoted ATP synthesis, and neuroprotection (51). Nevertheless, no evidence was provided that the NMDAR is translocated from the PM; therefore, other mechanisms for NMDAR transfer to mitochondria could be involved.

Notably, the GJ molecules **connexins** (Cx) have also been found at the mitochondria. Cx43 was found in mitochondria of human endothelial cells in a model of hyperhomocysteinemia, probably translocated from the PM (52). Later, functional hemichannels of Cx43 were located at the IMM of rat cardiomyocytes, with their C-terminal domains oriented toward the intermembrane space (53). Cx43 association with oxidative phosphorylation complex II was increased by an ischemia preconditioning (IP) protocol, and its translocation to mitochondria was mediated by the Hsp90-dependent TOM protein import system (54). Although these authors did not demonstrate the origin of Cx43 translocated to mitochondria, their short IP protocol (15 min), and the decrease of Cx43 at the cardiac intercalated discs (intercellular adhesion sites) after IP and Hsp90 inhibition, suggested that Cx43 was translocated from the PM. In a different work, Cx32 was also found at the IMM of hepatocytes, and its interactome contained several mitochondrial specific proteins, including the transporter sideroflexin-1 (55). With these observations, the authors claimed that the Cx32 interactome is a PM–mitochondria signaling nexus, that according to their bioinformatic analysis, it is involved in three networks: liver metabolic response to adipose-derived leptin, liver response to insulin/insulin receptor signaling, and interferon-gamma responsiveness/iron metabolism. Although these authors assumed somehow a direct contact between mitochondria and the PM, this was not demonstrated. Importantly, a role of the GJ in interorganelle communication has been hypothesized since some time ago based on the observation that vesicles lining the GJ of crayfish giant axon contain electron-opaque particles similar to junctional **innexons** (56).

On the other hand, it has also been reported that mitochondrial molecules can be found at the PM. Although some have attributed these observations to artifacts, some have suggested a relevant physiological role. Somatostatin-like protein 2 (SLP-2) localizes at the IMM as it contains a predicted mitochondrial targeting sequence, there it organizes cardiolipin-enriched domains facilitating the assembly of membrane-associated complexes. SLP-2 is involved in energy production, Ca^2+^ buffering, and apoptosis. Nevertheless, SLP-2 was initially found in PM lipid rafts, and in T cells, it was found that after minutes of activation, PM and mitochondrial SLP-2 populations coalesce near the IS (57). Although the transfer of SLP-2 from mitochondria to the PM was not demonstrated, the authors suggested that the occurrence of SLP-2 and other mitochondria molecules at the PM may reflect membrane exchange between these organelles. In this regard, the mitochondria-derived vesicles could be the means for such exchange (58), vesicles that demonstrate the dynamic nature of mitochondria membranes that occur in a steady-state and are induced by mitochondrial stress. Indeed, it has been found in exosomes that up to 10% of molecules are from mitochondrial origin (58).

More recently, we have found a direct interaction between mitochondria and the PM in rat cultured astrocytes (17), the same cells in which approximation and trapping of mitochondria were observed (see above) (49). While investigating the flux-independent and -dependent function of the NMDAR in these cells, we found that NMDA at acidic pH elicits mΔψ depletion, and the formation of caveolae in the vicinity of mitochondria that concur with electrodense structures that contact the PM and mitochondria (Fig. 3*B6*), effects that were accompanied by iCa^2+^ rise. These mitochondria were located 200 to 50 nm apart from the PM, but none contacted the PM plane. We did not observe structures at the interphase such as MACs, although perpendicular cisterna orientation to the PM, flattening of mitochondria membrane facing the PM, and bundles of filaments between mitochondria and the PM could be observed. We referred to these electrodense structures as PM–mitochondrial bridges because at the interior of mitochondria we observed dark spots that coincide with the electrodense structure's contacts, suggesting the entry of molecules into mitochondria (Fig. 3*B6*). Moreover, we also demonstrated mass transfer from the PM to mitochondria after NMDA treatment, further supporting the transfer of molecules. Notably, PM–mitochondrial bridges were observed only at the apical membrane of astrocytes and we found that the actin cytoskeleton is involved, not the microtubules, contrasting with previous findings described in the previous section with the same cells (49). In addition, mitochondria showed increased contacts with the ER and cell nucleus. These findings could be relevant for the response of astrocytes in physiological and pathological conditions, because it is well known that heavy glutamatergic activity at synapses generates synaptic cleft acidification, whereas in neuropathological conditions such as stroke, glutamate rises and pH drops even more dramatically. Interestingly, it has been described recently that astrocytes are capable of transferring functional mitochondria to neurons after stroke (59). This transfer was mediated by Ca^2+^, CD38, and ADP ribose signaling and resulted in enhanced neuronal survival. Although cellular details for such transfer were not described, it is possible that observations by Kolikova *et al.* (49) and ours show the early steps before mitochondria exit the cell (17, 49). In this regard, a striking mechanism of mitochondria transfer between cells through TNT has been reported and is briefly reviewed in the following section.

As mentioned above, in a very recent article with photoreceptors, it was described that mitochondria are tethered to the PM, with mitochondria of the neighboring cell also tethered (Fig. 3*B5*) (43), similar to observations made several decades ago in bat thyroid cells (Fig. 3*A1*) (28). These authors also found that these tethers are dynamic through development, that cristae openings of mitochondria are perpendicular to the PM, and that they can be opposed and aligned to cristae openings from mitochondria tethered in adjoining photoreceptors, an arrangement that was not observed in retinal pigment epithelium. In addition, they found that a heterozygous KO of OPA1, which plays a role in mitochondria fusion and cristae morphology and is associated with deficits in visual electrophysiology, altered mitochondria positioning but did not alter cristae alignment. With these findings, the authors proposed a form of mitochondria-mediated intercellular communication.

All in all, the interactions between mitochondria and the PM in different cells seem to present common features including stress as an effector that is linked with their dynamic nature, mass transfer of molecules, the benign physiological effects for cell metabolism and survival, as well as the involvement of the cytoskeleton. However, more studies are required to further elucidate the mitochondria–PM crosstalk in which molecular exchange seems to be critical. Notably, some of these observations indicate that vesicles are involved in PM–mitochondria interactions, shed from the PM and perhaps also from mitochondria, although this needs further investigation. Moreover, these works demonstrate that mitochondria–PM physical interactions play important metabolic roles in activated cells, which translate into the increase of cellular fitness, as early hypothesis had suggested, but also confirm the axiom that “location is function” because mostly subplasmalemmal mitochondria are involved. In addition, the findings in astrocytes reveal novel mechanisms of cell and mitochondria communication with the EC environment, which could be critical for tissue and organ function, in health and disease. Interestingly, the observation of apposed mitochondria to the PM in neighboring cells in photoreceptors and thyroid cells of hibernating bats suggests that this organization of mitochondria could be relevant for synchronized cells. Finally, mitochondria–PM physical interactions most probably have played a critical role in cell evolution because some mitochondria are located strategically to somehow sense the environment and respond metabolically, enabling the increase of cell fitness.

## Mitochondria transfer: TNTs

TNTs between cultured cells were discovered almost 15 years ago (60). These structures can mediate the transfer of cargo between cells. Here, we will briefly review the TNTs because they have been shown to mediate cell–cell mitochondria transfer. Although it has not been demonstrated that mitochondria–PM interactions are involved in this mechanism, the location of mitochondria at the entry of the TNT and the trespassing of the PM plane suggest it that way. More importantly, some involved molecules and mechanisms are common to mitochondria transfer through TNT and PM–mitochondria interactions, supporting that they could be related. Different authors have thoroughly reviewed TNTs recently; please refer to these works and references therein for further details on TNT induction, function, configuration, and other features not addressed here (14, 15, 61, 62, 63).

Initially, TNTs were found in pheochromocytoma cultured cell line (PC12) like nanotubular structures 50 to 200 nm wide and several cell diameters long that connected two cells (60). These structures have now been found in different cell types, and their genesis mechanism may differ. These structures present a continuous membrane to the connected cells, although other configurations have been described (14), they contain f-actin but not microtubules, as well as tubular or vesicular objects moving in one direction (at ∼26 nm/s). They transfer PM components, solutes, molecules, electrical signals, pathogens, or pathogenic proteins, actin, and organelles. This transfer is independent of endocytic, exocytic, or phagocytic events. TNTs have a high effectivity for mass transfer as 74% of cells connected presented mass exchange; however, they occur in a low percentage of cells (11%). As expected, one of the main questions this finding posed was whether TNTs also occur *in vivo*. Today, this question has been answered (64). TNTs have been also found to regulate cell differentiation during development, rescue metabolically compromised cells, help cell survival and proliferation, and improve the metabolism of transformed cells, some of these actions related directly to mitochondria transfer (14, 15, 61, 62, 63). Although initially TNTs were assumed to drive unidirectional mass transfer, it has been shown that bidirectionality may also occur, from the stressed cell to a healthy cell or in the opposite direction, and strikingly, TNT and mitochondria transfer can occur in an interspecies manner (15, 61). Interestingly, it has been shown that stress is the main elicitor of TNTs (hyperglycemia, acidified medium, endotoxin, serum starvation, H_2_O_2_, cytokines), similar to some of the mitochondria–PM interactions described above (14, 15, 61). Although the molecular mechanisms are still poorly understood, Miro1 and p53 have been involved, and importantly, Cx43 has been found critical for electric coupling and mitochondria transfer in a model of lung disease (65, 66), or after oxygen/glucose deprivation (67).

Other forms of mitochondria transfer do exist beyond TNT, further confirming the motile nature of mitochondria among cells in multicellular organisms. Mitochondria can be transferred through cell–cell fusion or its exocytosis in vesicles (61). Astonishingly, in unicellular organisms, the release of mitochondria to the EC milieu has also been reported. In the protozoan *Tetrahymena*, 5 to 20% of total mitochondria can be released from the cell after activation through the clustering of glycosylphosphatidylinositol-anchored PM proteins (68). Mitochondria release in this protozoan was not related to the death process and required iCa^2+^ rise. Interestingly, Hsp60 was redistributed from mitochondria to the PM after activation, and mitochondria release was found to increase cell survival. These authors hypothesized that mitochondria release is deeply rooted in evolution and that it may have a relevant biological significance for the cell. This idea is consistent with the increase of cellular fitness by mitochondria transfer from cell to cell through TNTs or other means, as it has been shown in rodents after stroke (59). Moreover, it was reported recently that functional mitochondria can be found in the blood of healthy and diseased patients, raising questions on the frequency and relevance of mitochondria motility for multicellular physiology and health (13). These observations together with the properties of mesenchymal stem cells as mitochondria donors, capable to rescue metabolically stressed cells, have led to the idea that mitochondria transfer could be used as therapy for some diseases in which mitochondria function is compromised (69).

Taken together, these findings first demonstrate that mitochondria are not subdued organelles of the cell (3), as it has been considered for decades, as they can jump out from or into cells under certain circumstances. On the other hand, regarding the still obscure mechanism for mitochondria transfer, through TNT, cell fusion, vesicles, or extrusion, mitochondria–PM interactions are expected to be critical for these processes that improve cell fitness. However, this still needs to be investigated, but the common role of Cx, stress, the cytoskeleton, and mitochondria metabolic compromise is consistent with this idea.

## Discussion and conclusions

Although the findings described above clearly show that some mitochondria are able to establish specific interactions with the PM (with or without direct physical contact), several questions remain to be investigated. First, it must be noted that the interactions described in yeast and mammalian cells seem to diverge. In yeast, a single molecule has been found to contact both organelles, Num1, that contacts both the PM and the OMM (19, 21), generating an attachment of at most dozens of nanometers. The PM tubular invaginations observed by Keckler *et al.* (21) that span into the cytoplasm that are hundreds of nanometers long enable that such a short distance is achieved (Fig. 2, *B* and *C*). In contrast, PM–mitochondria interactions in mammalian cells at intercellular contact sites seem to be more diverse and complex, most probably with other molecular players involved in the formation of structures, contact sites, or MACs. Similar structures have not been observed in yeast, although, the participation of homologous or analogous proteins to Num1 or Mdm36 from yeast cannot be ruled out *a priori*. Further genetic and molecular studies in mammalian cells are required to elucidate the molecular mechanisms and players involved in physiological and pathological conditions. In addition, MACs have not been observed associated with tubular invaginations of the PM, as mitochondria in yeast, although in cardiac myocytes, where apparently no MACs are present, similar tubular invaginations radiating from caveolae toward mitochondria have been shown (Fig. 3*A5*) (18). Moreover, caveolae have been found proximal to subplasmalemmal mitochondria in epithelial cells and astrocytes under different conditions and could be the means to achieve direct contact between mitochondria and PM domains, as our findings support (Fig. 3, *A4* and *B6*) (17). More research is required to disentangle the role of PM invaginations for these interactions. Interestingly, in TEM images of mammalian cells, mitochondria are consistently located 50 to 300 nm from the PM plane. This observation hints that somehow the cell does not allow mitochondria to move closer to the PM, perhaps the cortical actin cytoskeleton or mitochondria anchoring, although this needs to be tested. Nevertheless, under particular metabolic conditions, large areas of mitochondria can be directly tethered to the PM (Fig. 3, *A1* and *B5*) (28, 43), further supporting that metabolic activity is involved in determining mitochondria relationship with the PM and suggesting that cell synchronization is related with this phenotype. In hibernating animals, the metabolic activity is low in tissues and organs, whereas in photoreceptors, their high energy consumption requires strategies that help maintain homeostasis. In both cases, it seems feasible that the sharing of metabolites and resources among mitochondria may help in this endeavor, through mitochondria-mediated intercellular communication, as has been suggested recently (43). This in turn could assist to keep the activity of cells synchronized and visual acuity in the case of photoreceptors. In this regard, it is notable that the organization of cisternae perpendicular to the PM has been observed in almost all phenotypes of PM–mitochondria interactions (Fig. 4), even when mitochondria from neighboring cells are involved (Fig. 3, *A1*, *A3*, *A5*, *A6*, *B1*–*B3*, *B5*, and *B6*). This organization could support an efficient putative sharing of metabolites and resources (39). More experiments are required to clarify how cell and organism metabolisms regulate PM–mitochondria interactions and vice versa.

One feature of these interactions in mammalian cells seems to be clear: they are induced or favored by cell stressors. This feature further confirms the role of metabolism in the establishment of these interactions, but also demonstrates that they may be dynamic, as has been shown in yeast (22) and through development by different groups (41, 43). This property could offer an alternative scheme for the observations made in mammalian cells. It is possible that, depending upon the tissue and cell, these interactions can be established *de novo* as in astrocytes or cardiomyocytes, if they are not already somehow established through unobserved structures and/or molecules, and deconstructed. If so, the mitochondria–PM interactions in yeast and mammalian cells could be different stages in the process of establishing new interactions, which can reach a more or less permanent state, as suggested by observations in epithelial and neuronal cells with adhesion sites and MACs involved. On the other hand, it is also possible that different types of interactions through distinct molecular players are established in mammalian cells. More work is required to test these possibilities, to establish the role of cellular stress and to investigate whether mitochondria release is conserved as it has been observed in the protist *Tetrahymena*. In Figure 4, a schematic representation of the complexities in PM–mitochondria interactions is shown.

It is intriguing that Cx have been identified as molecules transferred from the PM to mitochondria, that they also participate in the transfer of mitochondria from cell to cell through the TNT, and that they have been hypothesized to mediate interorganelle communication (14, 56). These findings suggest a relevant role of Cx in mitochondria mobility and function; however, this still needs to be elucidated.

A relevant issue regarding the transfer of molecules with transmembrane domains from the PM to the mitochondria is the topology of their intracellular and EC domains. It has been shown for some transferred molecules from the PM to the IMM that they keep their cytoplasmic side toward the intermembrane space. This orientation has tremendous implications for their function because, in the case of Cx, it could implicate that the same cytoplasmic signals that regulate GJ opening at the PM could also regulate the opening of Cx in the mitochondria intermembrane space, provided that such signals access this compartment. It is tempting then to speculate that the EC milieu could in principle form a continuum from the EC space to the mitochondria matrix, limited by the GJ pore size and the access to the mitochondria intermembrane space; however, this hypothesis requires to be tested. The mechanism responsible for PM molecule translocation to mitochondria has not been fully elucidated. In the case of Cx43, the mechanism involved the Hsp90-dependent TOM protein import system (54), and vesicles seem to play a role. However, more research is required to confirm this and further disentangle the biophysics and biochemistry of this mechanism and its consequences, as well as, the role of molecular translocation between the PM and mitochondria. Intriguingly, the NMDAR located in neuronal mitochondria seems to have the opposite topological orientation to the PM receptor because the analogous of cytoplasmic glutamate modulated its function (51), and at the PM receptor, the glutamate-binding domain is EC.

All in all, mitochondria–PM interactions reviewed here indicate that some mitochondria have a strategic location within the cell that allows them to sense the EC milieu through molecular diffusion within the cytoplasm, mass exchange, physical interactions, and/or the assembly of structures (Fig. 4). This in turn enables the adaptation of mitochondria that at the same time promotes cell fitness. These interactions seem to comprise a novel emerging pathway of intracellular communication that challenges the extended conception, considering mitochondria as obligate, subdued organelles of the eukaryotic cell, subjugated to cell physiology and the intracellular environment with scarce or no interaction with the EC milieu. This conception springs from the idea that the cell host forms an interphase that keeps mitochondria isolated and protected from the external milieu. However, the findings reviewed here suggest that some mitochondria subsets directly interact with the PM and the EC milieu. This feature is consistent with the proposed origin of mitochondria and the diversity of integration pathways followed through evolution by this organelle in distinct organisms (4). In this regard, the EC milieu sensing by mitochondria could have provided a selection pressure that cooperated to shape the integration of early mitochondria to the eukaryotic cell when it diversified, leading to the development of strategies that improve or make possible their viability (according to phenotype, location, milieu, etc), resulting in the diversity of phenotypes observed in mitochondrial DNA lengths, number of genes, and organization (4).

This pathway of intracellular communication along with the strategic location of some mitochondria enables a fast metabolic response by the archaic α-proteobacteria that adapted to intracellular living, modulating its mobility, physiology, and metabolism that ultimately impact cellular responses, survival, and tissue health. However, the implications of these interactions for cell metabolism, physiology, pathology, and evolution still need to be elucidated. Finally, the involvement of vesicles in these interactions would profile a novel emerging vesicular intracellular traffic pathway that requires further investigation, including the putative role of mitochondria-derived vesicles. Nevertheless, considering the role of mitochondria, it is possible to speculate that mitochondria–PM interactions must be critical for health and disease but also must have been pivotal for eukaryotic and multicellularity evolution when cell–cell communication and integration acquired an unprecedented relevance. Hopefully, research in the following years will provide answers to questions raised by PM–mitochondria interactions.

## Conflict of interest

The author declares that he has no conflicts of interest with the contents of this article.
